# Open reduction and internal fixation of the tibial avulsion fracture of the posterior cruciate ligament: which is better, a hollow lag screw combined with a gasket or a homemade hook plate?

**DOI:** 10.1186/s12891-022-05096-0

**Published:** 2022-02-11

**Authors:** Hongfei Qi, Yao Lu, Ming Li, Cheng Ren, Yibo Xu, Teng Ma, Qian Wang, Kun Zhang, Zhong Li

**Affiliations:** Department of Orthopaedics and Trauma, Hong Hui Hospital, Xi’an Jiaotong University College of Medicine, No. 555, East Youyi Road, Xi’an, 710000 Shaanxi China

**Keywords:** Homemade hook plate, Internal fixation, PCL tibial avulsion

## Abstract

**Objective:**

To compare the clinical results of homemade hook plates and hollow lag screws combined with spacers in the treatment of posterior cruciate tibial ligament avulsion fractures.

**Materials and methods:**

This was a retrospective clinical cohort study that included 64 patients with PCL tibial avulsion fractures. Thirty-two of them were fixed with a homemade hook plate (hook plate group), and 32 were fixed with a hollow lag screen combined with a gasket (hollow lag screen group). By reviewing the medical record data and follow-up results, the operation time, postoperative drainage, fracture healing time, surgical complications, knee mobility, recovery of joint function, and whether postoperative gastrocnemius muscle strength changed in the two groups were compared.

**Results:**

All patients had successful wound and fracture healing. No adverse events, such as bone nonunion, infection, wound haematoma, or joint stiffness, occurred in either group. There were no patients with decreased gastrocnemius muscle strength in either group. Internal fixation failure occurred in 2 cases in the hollow lag screen group but not in the hook plate group. There were no significant differences between the two groups in terms of operative time, postoperative drainage, fracture healing time, knee mobility at the last follow-up, or Lysholm score.

**Conclusion:**

It is safe and effective to use a homemade hook plate to fix PCL tibial avulsion fractures through an inverted L-shaped posterior medial approach. A homemade hook plate may have potential advantages over a hollow lag screen combined with gasket fixation.

## Background

The posterior cruciate ligament (PCL) is the primary constraint of posterior tibial translation at 30 and 90° of flexion. PCL injury may lead to changes in the stability of the knee joint, causing the tibia to move backward, impeding the ability to keep the back of the knee joint straight, and long-term, this will induce degenerative changes in the knee joint [1]. In contrast to anterior cruciate ligament (ACL) injuries, PCL injuries mainly manifest as avulsion fractures of the attached ligament [2]. Avulsion injuries are different from other PCL injuries because they are easily diagnosed by standard X-ray films, and bone fragments can be clearly seen.

Arthroscopy-assisted reduction and fixation of the fracture has the advantages of less trauma and rapid recovery, and it has become the first choice for sports injury doctors [3–5]. Due to the unbalanced development of medical technology, most primary medical institutions cannot master or develop arthroscopic technology. Because of the long learning period and high requirements, open reduction and internal fixation are often the only available choices. Fortunately, biomechanical studies have also demonstrated comparable results of screw fixation using open or arthroscopic means [6]. At present, a variety of internal fixation materials are used for PCL injury fixation, including hollow lag screens, steel wires, absorbable screens, suture anchors and straddle nails [5, 7, 8]. Veselko et al. [9] reported that good clinical effects can be observed by using a hollow lag screw combined with gasket fixation. A 1/3 tubular plate is transformed into a hook plate, which can be used for the fixation of PCL damage. It has the advantages of reliable fixation, simple operation and low cost [9]. To compare the efficacy of the above two fixation groups, we retrospectively analysed the clinical data and postoperative recovery of patients with PCL tibial avulsion fractures who underwent surgery at the Honghui Hospital of Xi’an Jiaotong University from January 2018 to January 2021.

## Data and methods

Inclusion criteria: 1. Diagnosed with PCL tibial avulsion fractures; 2. Diagnosed with Meyers McKeever II and III; 3. Age > 18 years; 4. Good joint function before the knee injury, 4. Complete follow-up data.

Exclusion criteria: 1. Patients with anterior cruciate ligament and collateral ligament injuries; 2. Patients with fractures of the distal femur or proximal tibia; 3. Patients with severe complications and inoperability; 4. Nondisplaced fracture fragments or fragment displacement < 10 mm; 5. Time from injury to operation> 2 weeks; 6. Patients with preoperative joint dysfunction or a history of knee joint trauma; 7. Open fractures.

A total of 64 patients were enrolled in this study, 32 of whom were treated with hollow lag screens, including 24 males and 8 females; 32 patients were treated with hook plates, 20 males and 12 females. In the hollow lag screen group, the mean age was 33.4 ± 10.3. There were 19 patients with sports-related injuries and 13 patients with traffic accidents. According to the Meyers-McKeever classification, there were 5 cases of type II and 27 cases of type III. Eight cases were complicated with meniscus injury, and the average time from injury to operation was 5.7 ± 2.1 days. In the hook plate group, the age was 31.6 ± 11.7, 17 cases were caused by sports-related injuries, and 15 cases were caused by traffic accidents. According to the Meyers-McKeever classification, there were 7 cases of type II and 25 cases of type III. Ten cases were complicated with a meniscus injury, and the average time from injury to operation was 4.9 ± 1.8 days. There was no significant difference (*P* > 0.05, Table 1) between the two groups of patients in terms of sex, age, cause of injury, type of fracture, or time from injury to operation. This study was approved by the ethics committee of the Honghui Hospital of Xi’an Jiaotong University, and all patients included in the study signed an informed consent form.

**Table 1:** Comparison of the baseline data between the two groups

Variable	Hollow lag screen group (***n*** = 32)	Hook plate group (*n* = 32)	T/X^**2**^	***P*** value
Gender (female/male)	8/24	12/20	1.164	0.281
Age (yr)	33.4 ± 10.3	31.6 ± 11.7	0.653	0.516
Meyers-McKeever classification			0.410	0.522
II	5	7	0.103	0.749
III	27	25		
Mechanism of injury			0.254	0.614
Sports-related	19	17		
Traffic accidents	13	15		
Mean time from injury to operation (d)	5.7 ± 2.1	4.9 ± 1.8	1.636	0.167

### Surgical treatment

#### Hook plate group

**Fig. 1:**
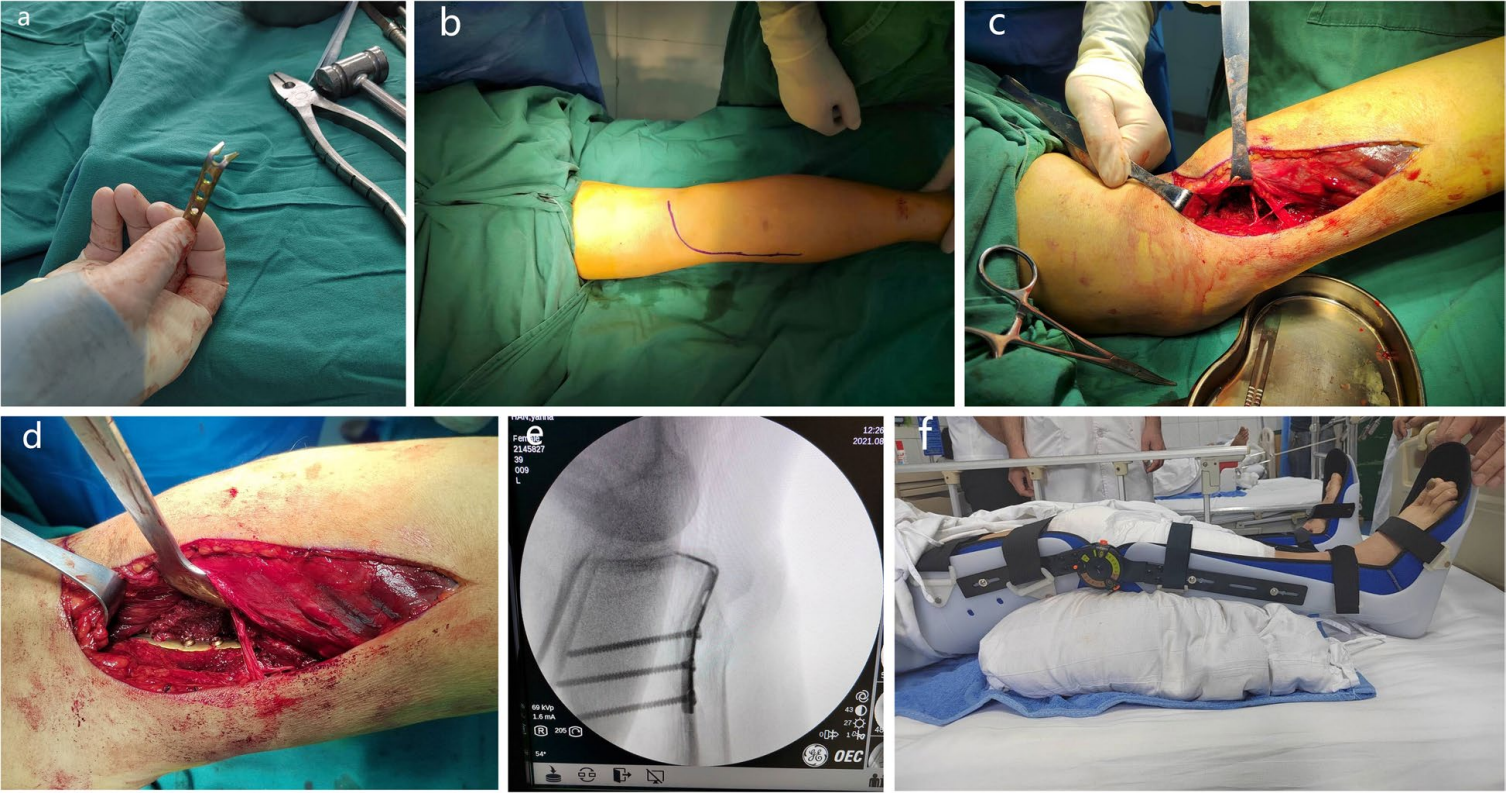
Intraoperative and postoperative conditions of the tibial avulsion fracture of the PCL fixed by a homemade hook plate. **a** Hook plate made by cutting a 1/3 tubular plate; **b**-**c** the L-shaped surgical incision; **d** fixation of the fracture with the hook plate and screw; **e** C-arm fluoroscopy to confirm the fracture fixation; **f** the adjustable brace that was worn on the first day after the operation

The hook plate was made by cutting a 5-hole 1/3 tubular plate. The proximal 3-hole segment was retained, and Kirschner scissors were used to cut the fourth hole at an angle of 30° with the edge of the plate (the cutting length of the steel plate was determined according to the fracture fragments). Then, wire pliers were used to bend the “sharp teeth” into a hook (Fig. 1a). After successful anaesthesia, the patient was placed in the prone position, the affected limbs were routinely disinfected, the sterile operation sheet was applied, and the air bag tourniquet was put on.

An L-shaped surgical incision with a length of approximately 8–10 cm from the posteromedial side of the knee joint was made. Starting from the transverse lines of the popliteal skin, the incision was transverse to the medial edge of the gastrocnemius muscle, turned directly to the medial side of the leg and extended to the distal end (Fig. 1b). The skin, subcutaneous tissue and fascia were cut layer by layer, and the medial head of the gastrocnemius was pulled outward to protect the blood vessels and nerve bundles of the popliteal fossa (Fig. 1c).

After opening the joint capsule, the knee joint was bent, and we peeled off and exposed the PCL and the avulsion fracture fragments. We carefully cleaned away the congestion and blood clots at the broken end of the fracture, rinsed the joint cavity and reset it, clamped it with the Kirschner wire and point forceps, and fixed the fracture with the hook plate and screw (Fig. 1d). Passive knee movement on the operating table was used to ensure good movement, a stable fracture, satisfactory fracture reduction under C-arm fluoroscopy, and a good internal fixation position (Fig. 1e). After washing the wound repeatedly with a large amount of normal saline, we counted the instrument accessories, left a drainage tube in the wound, stopped all bleeding, reset the skin margin, closed the flap layer by layer, bound the wound with sterile accessories, and loosened the air bag tourniquet.

#### Hollow lag screen group

The patient was placed in a prone position, with the knee joint flexed at 30°, and an arc surgical incision was made the same as in the hook plate group. The skin and subcutaneous tissue were cut layer by layer, and the fascia located at the medial edge of the gastrocnemius was cut longitudinally to open the gastrocnemius and semimembrane muscles. The medial head of the gastrocnemius muscle was pulled to the outside, the popliteal oblique ligament and posterior joint capsule tissue were cut, and the posterior angle of the medial meniscus was exposed. At this time, the tibial attachment of the posterior cruciate ligament could be touched. After removing the soft tissues and blood clots stuck between the fragments, we temporarily fixed the bones with Kirschner wires. We properly drilled holes according to the size of the avulsion fragments, placed the gasket and hollow lag screen fixation after measurement, and confirmed the position of the fracture with C-arm fluoroscopy. After the reduction was satisfactory, the wound was washed and sutured layer by layer.

### Postoperative treatment

Antibiotics were routinely used for 24 h after surgery, and an adjustable brace was worn on the affected limb to fix the knee joint and maintain flexion for 30° for 1–2 weeks (Fig. 1f). On the first day after surgery, exercises such as hooking and lifting the legs were performed. On the second day after surgery, the drainage tube was removed from the wound. After 1–2 weeks, knee joint range of motion (ROM) exercises were gradually started. X-ray was performed every 2–4 weeks to observe the fracture healing and limb function. Patients could walk with crutches 4 weeks after the operation and with complete weight-bearing 8 weeks after the operation.

### Efficacy evaluation

The operation time, postoperative drainage, postoperative complications, fracture healing time, knee range of motion, and knee joint function were compared between the two groups. Postoperative complications included nonunion, infection, wound haematoma, joint stiffness, and failure of internal fixation. Knee X-rays were completed every 2–4 weeks after the operation to observe the fracture reduction and healing. At the last follow-up, the posterior drawer experiment was performed to evaluate the laxity of the knee joint, which was classified as Grade I, Grade II, and Grade III. At each follow-up, the patient was instructed to lie prone on the checklist with a standard goniometer to measure the ROM, and the Lysholm score was used to evaluate the knee joint function.

### Statistical analysis

Data analysis was performed using SPSS version 13.0 (SPSS Inc., Chicago, IL, USA). Continuity variables are represented by the mean ± standard deviation. Independent sample t-tests were used for comparisons between groups. The X^2^ test was used to compare the count data between the two groups. *P* < 0.05 was considered to be statistically significant.

## Results

The patients included in this study completed at least 12 weeks of follow-up after surgery. All patients had primary healing of the wound after surgery. There were no adverse events, such as bone nonunion, infection, wound haematoma or joint stiffness, in either group. None of the patients experienced a decrease in gastrocnemius muscle strength. Among them, 2 patients in the hollow lag screen group had X-rays in the 6th week after surgery that indicated displacement of the fracture mass (internal fixation failure). The second stage was fixed with a hook plate, and the fracture was finally healed.

The operation time in the hollow lag screen group was 58.5 ± 10.4 min, and the operation time in the hook plate group was 61.2 ± 1.1 min. There was no significant difference in the operation time between the two groups (*P* = 0.319). The postoperative drainage volume of the hollow lag screen group was 100.9 ± 21.3 ml, the hook plate group was 98.4 ± 22.5 ml, and there was no significant difference between the two groups (*P* = 0.651). According to the results of the X-ray review, the mean fracture healing time of the hollow lag screen group was 11.65 ± 2.31 ml, the hook plate group was 11.32 ± 2.17 ml, and thus the fracture healing time of the two groups was basically the same (*P* = 0.558). At the last follow-up, the patients in both groups had completely healed.

The ROM was 132.56° ± 8.39° in the hollow lag screen group and 135.21° ± 6.37° in the hook plate group. There was no significant difference between the two groups (*P* = 0.161). The posterior drawer test of all patients was negative, and the knee joint was stable. The Lysholm score of the hollow lag screen group was 95.07 ± 4.32, and that of the hook plate group was 95.63 ± 3.46. There was no significant difference in the Lysholm score of the two groups at the last follow-up (*P* = 0.569, Table 2).

**Table 2:** Comparison of postoperative and follow-up data between the two groups

Variable	Hollow lag screen group (*n* = 32)	Hook plate group (*n* = 32)	T/X^**2**^	*P* value
Operation time (min)	58.5 ± 10.4	61.2 ± 11.1	−1.004	0.319
Postoperative drainage (ml)	100.9 ± 21.3	98.4 ± 22.5	0.456	0.651
Fracture union time (week)	11.65 ± 2.31	11.32 ± 2.17	0.589	0.558
Knee flexion (°)	132.56 ± 8.39	135.21 ± 6.37	−1.423	0.161
Lysholm score	95.07 ± 4.32	95.63 ± 3.46	−0.572	0.569
Internal fixation failure	2	0	0.516	0.472

**Fig. 2:**
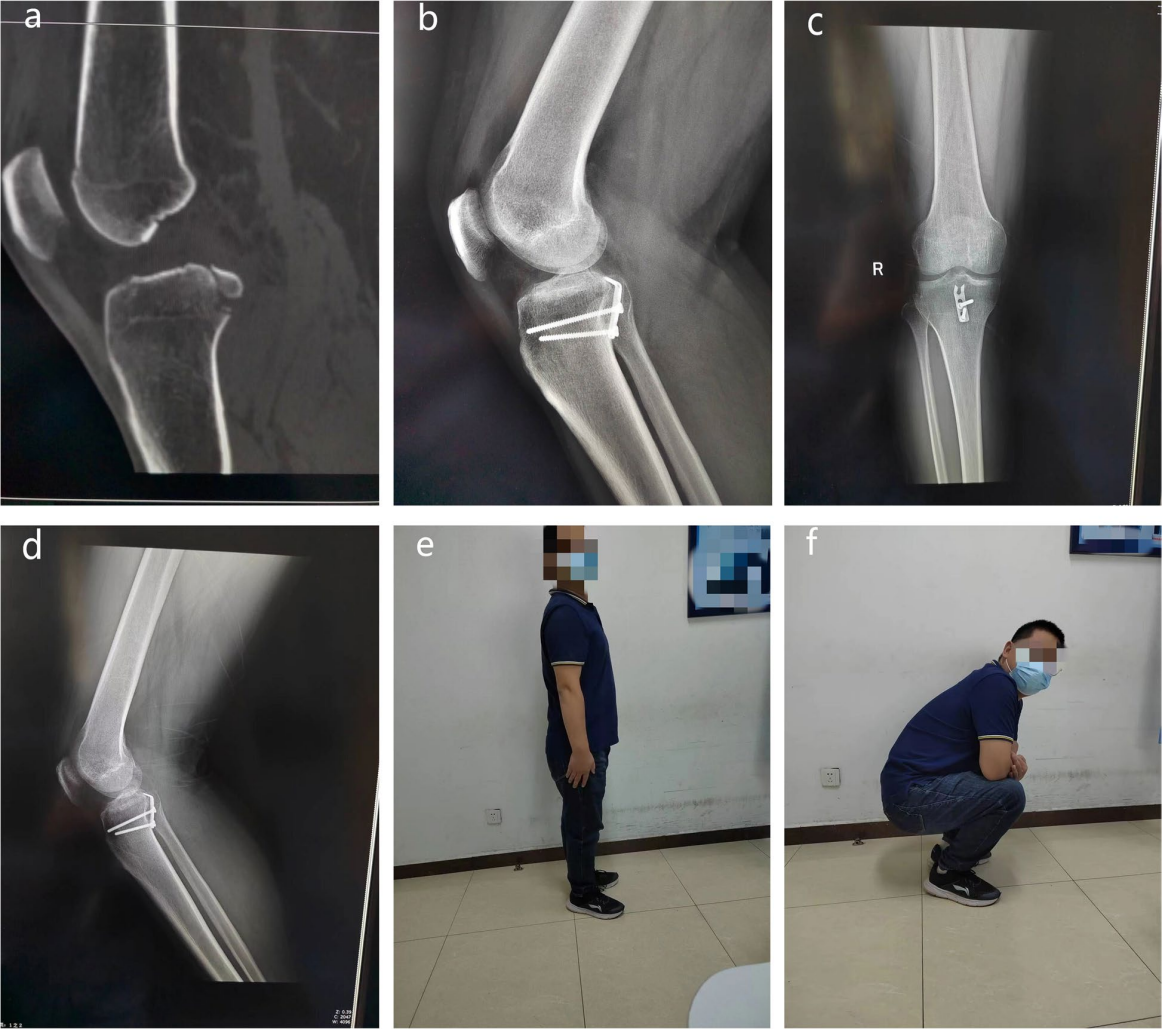
A 32-year-old male presented with an avulsion fracture of the PCL of the right knee. **a** CT images of the right knee showed PCL avulsion fracture and fragment displacement. **b** Lateral X-ray film of the knee joint on the first day after the operation. **c-d** X-ray showing bone healing 3 months after the operation. **e-f** Photos showing that the patient’s right knee function was good 6 months after the operation

**Fig. 3:**
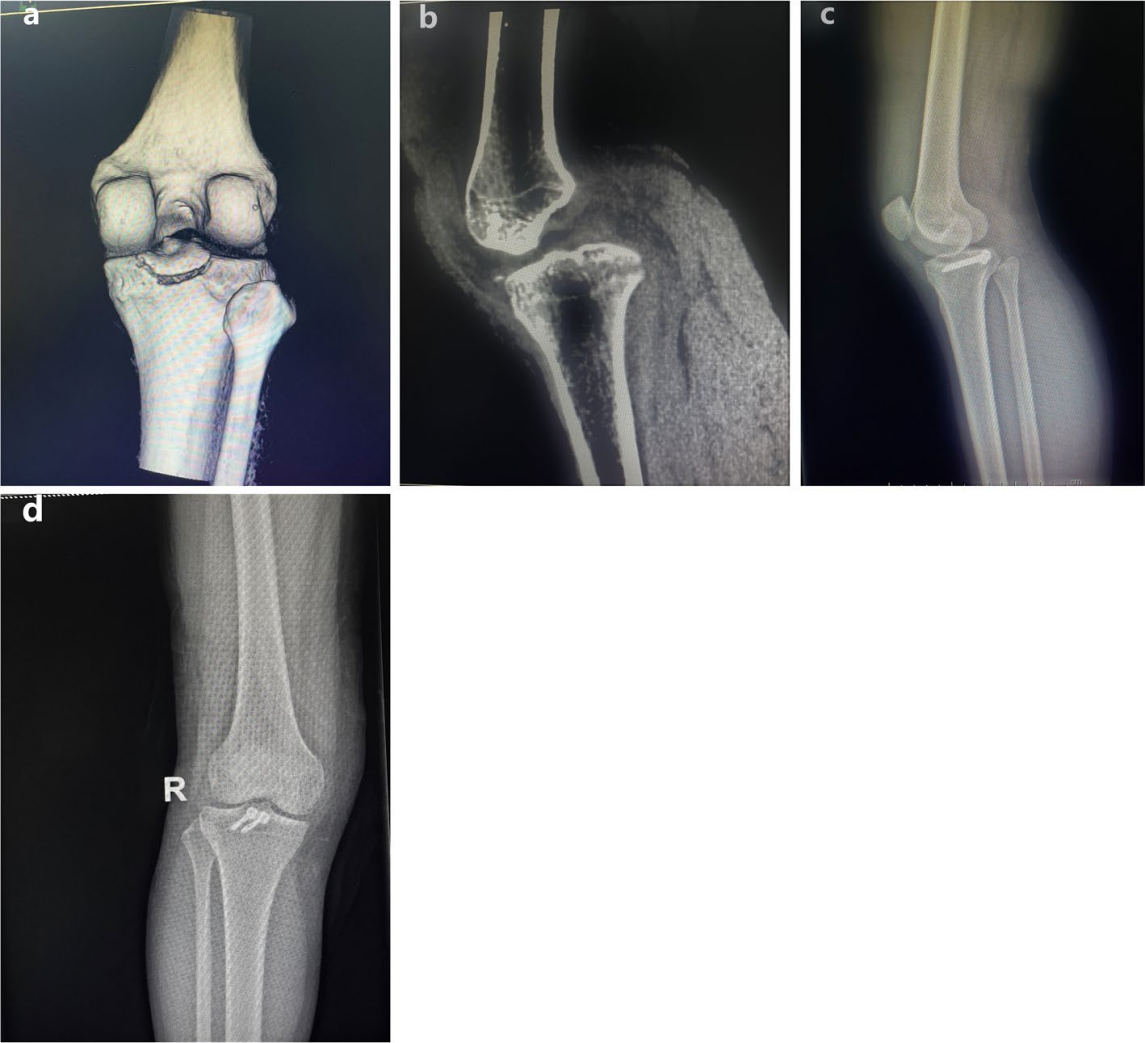
**a**,**b** Preoperative CT showed an avulsion fracture of the posterior cruciate ligament. **c**,**d** A hollow lag screen combined with a gasket operation. X-ray films of the patient’s knee joint on the 2nd day after the operation

Typical cases of the hook plate group and the hollow lag screen group are shown in Fig. 2 and Fig. 3 respectively.

## Discussion

The main purpose of this study was to determine whether a homemade hook plate used in tibial avulsion fractures of the posterior cruciate ligament can effectively restore the stability of the knee joint. Recent studies [10, 11] have shown that hook plates have a good clinical effect in avulsion fractures of the posterior cruciate ligament. However, no study has compared this approach with the hollow lag screen approach; which has a better effect for treating avulsion fracture of the posterior cruciate ligament? The hook plate technology has the advantages of a simple operation, low postoperative complications, good postoperative knee function recovery, and a low cost. All patients in this study achieved good knee joint function after the operation, and no patients had complications such as internal fixation loosening or fracture displacement. This shows that homemade hook plate treatment of tibial avulsion fractures of the posterior cruciate ligament is a technique worth promoting.

PCL wounds account for about20% of total ligament damage of the knee [12]. They commonly occur in traffic accidents and people who participate in contact sports are particularly vulnerable to PCL injuries [13, 14]. The avulsion fractures of posterior cruciate ligament are classified by Meyers-McKeever into three types: type I has no displacement of fracture fragments; type II has a little displaced posterior edge with an intact anterior cortex acting as a hinge; and type III has a completely displaced void of all bone connections. Nonoperative therapy may be recommended if the avulsed fragment is not displaced, but surgical reduction and fixation should be considered in cases of type II and III fractures [15, 16]. It has been claimed that surgical treatment of type I fractures can restore PCL tension, reduce immobilization time, and enhance knee joint function after surgery as compared to conservative treatment [17–19]. Surgical therapy is often reserved for type II and III fractures, particularly in young patients with vigorous lower limb function. Articular cartilage degradation and quadriceps atrophy are possible side effects of conservative therapy [20, 21]. Early repair of the PCL can effectively avoid these situations.

The optimum option is arthroscopic surgery, which has the advantage of being minimally invasive. Metal screws, sutures, steel wires, and anchors are popular surgical fixation materials [9, 22–25]. Although arthroscopic repair is less invasive and can treat associated ailments such as meniscus and synovial problems at the same time, it is a technically challenging procedure with a steep learning curve and specialized equipment. The use of arthroscopic procedures for reduction and fixation is more difficult than open surgery. As a result, many primary medical facilities may find ORIF more practical. The classic posterior median or S-shaped incision for ORIF requires separating or cutting off part of the medial head of the gastrocnemius muscle, which might lead to gastrocnemius weakness after the procedure [26]. Furthermore, this method has a high risk of vascular and nerve injury in order to better expose the fracture. The open posteromedial technique avoids nerves and blood arteries, allowing for a better view of the broken end of the bone, and enables for complete fracture reduction and successful fracture fragment fixation [27]. The incision has the advantages of being a straightforward procedure with a small incision and minimal scars. Even if a scar remains after this minor incision, it will not cause knee stiffness. All cases in our study adopted the open posteromedial approach, and no patients developed gastrocnemius weakness after surgery, which is consistent with previous studies.

With the progression of clinical practice, some new situations have been discovered. Although the hollow lag screw can fix large fractures, for small or comminuted fractures, there may be a risk of redisplacement or unstable fixation. In spite of the fact that stay focuses and non-absorbable sutures are utilized for tendon remaking surgery, they may not be able to effectively fix the fracture, and early postoperative functional movement may cause the fracture to be displaced again [28]. Shino et al. [29] reported that a single cannulated screw or sutures and a pullout button achieved good results in the fixation of avulsed fractures of the posterior cruciate ligament. Joshi et al. [30] reported that 14 cases of PCL avulsion fracture patients were treated with cancellous screws combined with sutures. They found that the process of suture fixation was difficult. The suture must pass through the bone tunnel to the inside of the tibial tubercle and be tied, which requires cutting and loosening of the bone [31]. In 2003, Veselko et al. [9] proposed that hollow hysteresis screws and gaskets could be used in the treatment of tibial PCL avulsion fractures. Using this method, it can be fixed under pressure during the operation. However, the l gasket scatters the stress of the screw to the broken fragments, which makes the gasket incapable to effectively control the rotational stability of the broken fragments.

In our study, a homemade hook plate was used to fix the tibial PCL avulsion fractures. In clinical applications, we found that this method has the advantages of a larger fixation range, simple and convenient operation, simple steel plate production, and low cost. First, during the operation, the avulsion fracture of the PCL tibia can be exposed under direct vision, which can avoid re-embedding the surrounding soft tissue into the fracture site and reduce the risk of non-union. Second, the plate can compress the fracture evenly and guarantee close contact of the broken ends. Since the lower surface of the hook is in face-to-face contact with the fracture, cutting damage is avoided. In addition, the homemade hook plate can also fix small or even comminuted fractures. The large contact area of the hook helps to control the rotation of the crack. The direction of screw insertion is basically perpendicular to the surface of the posterior tibia, and the screw is inserted into normal cortical bone. Finally, this fixation method is more reliable, and the screw will not loosen or retract with flexion and extension of the knee joint. The knee joint is dominated by cancellous bone, with an abundant blood supply, and fracture healing is rapid.

The results of our study showed that during treatment of tibial avulsion fracture of the posterior cruciate ligament, a hollow lag screw combined with gasket fixation and a homemade hook plate fixation, the two groups of patients had similar operation times, postoperative drainage, fracture union time, joint function at the last follow-up time, and Lysholm scores at the last follow-up time. There was no statistically significant difference. However, two patients in the hollow lag screw group experienced internal fixation failure after surgery. Although this difference was not statistically significant, no patients with homemade hook plates experienced internal fixation failure, which may imply that the fixation effect of homemade hook plates is more reliable. Of course, the efficacy of homemade hook placement needs to be verified by a larger sample in randomized controlled trials.

## Conclusion

The homemade hook plate is an effective way to fix displaced PCL avulsion fractures. Through this method, patients can obtain good clinical, imaging and functional results. At the last follow-up, all patients obtained good knee function, and the back drawer test of the knee joint was stable. In addition, the homemade hook plate also had good cost-effectiveness. None of the patients had any complications, such as gastrocnemius weakness, joint stiffness or bone nonunion.

## Data Availability

The data that supported the findings of this study are available on request from the corresponding author. The data is not publicly available due to privacy or ethical restrictions.
